# Modulation of Cognitive and Emotional Control in Age-Related Mild-to-Moderate Hearing Loss

**DOI:** 10.3389/fneur.2018.00783

**Published:** 2018-09-19

**Authors:** Artyom Zinchenko, Philipp Kanske, Christian Obermeier, Erich Schröger, Arno Villringer, Sonja A. Kotz

**Affiliations:** ^1^International Max Planck Research School on Neuroscience of Communication (IMPRS NeuroCom), Leipzig, Germany; ^2^Department of Neuropsychology, Max Planck Institute for Human Cognitive and Brain Sciences, Leipzig, Germany; ^3^Department Psychologie, Ludwig-Maximilians-Universität München, Munich, Germany; ^4^Chair of Clinical Psychology and Behavioral Neuroscience, Faculty of Psychology, Technische Universität Dresden, Dresden, Germany; ^5^Department of Social Neuroscience, Max Planck Institute for Human Cognitive and Brain Sciences, Leipzig, Germany; ^6^Institute of Psychology, University of Leipzig, Leipzig, Germany; ^7^Department of Neuropsychology and Psychopharmacology, Faculty of Psychology and Neuroscience, Maastricht University, Maastricht, Netherlands

**Keywords:** ERPs, aging, hearing loss, cognitive conflict, emotional conflict, affective modulation, executive control

## Abstract

Progressive hearing loss is a common phenomenon in healthy aging and may affect the perception of emotions expressed in speech. Elderly with mild to moderate hearing loss often rate emotional expressions as less emotional and display reduced activity in emotion-sensitive brain areas (e.g., amygdala). However, it is not clear how hearing loss affects cognitive and emotional control mechanisms engaged in multimodal speech processing. In previous work we showed that negative, task-relevant and -irrelevant emotion modulates the two types of control in younger and older adults without hearing loss. To further explore how reduced hearing capacity affects emotional and cognitive control, we tested whether moderate hearing loss (>30 dB) at frequencies relevant for speech impacts cognitive and emotional control. We tested two groups of older adults with hearing loss (HL; *N* = 21; mean age = 70.5) and without hearing loss (NH; *N* = 21; mean age = 68.4). In two EEG experiments participants observed multimodal video clips and either categorized pronounced vowels (cognitive conflict) or their emotions (emotional conflict). Importantly, the facial expressions were either matched or mismatched with the corresponding vocalizations. In both conflict tasks, we found that negative stimuli modulated behavioral conflict processing in the NH but not the HL group, while the HL group performed at chance level in the emotional conflict task. Further, we found that the amplitude difference between congruent and incongruent stimuli was larger in negative relative to neutral N100 responses across tasks and groups. Lastly, in the emotional conflict task, neutral stimuli elicited a smaller N200 response than emotional stimuli primarily in the HL group. Consequently, age-related hearing loss not only affects the processing of emotional acoustic cues but also alters the behavioral benefits of emotional stimuli on cognitive and emotional control, despite preserved early neural responses. The resulting difficulties in the multimodal integration of incongruent emotional stimuli may lead to problems in processing complex social information (irony, sarcasm) and impact emotion processing in the limbic network. This could be related to social isolation and depression observed in the elderly with age-related hearing loss.

## Introduction

Healthy aging is often accompanied by a progressive decline in hearing capacity or even hearing loss [HL; (1)]. The prevalence of HL is close to 40% in those of 65 years or older (2) and mild-to-moderately severe sensorineural HL affects up to 33% of the world's adult population (3, 4). Hearing loss modulates the processing of acoustic information in the auditory cortex as well as along the ascending auditory pathways. For instance, Alain et al. (5) used magnetoencephalography to measure auditory evoked fields (AEFs) in a task where participants listened to complex sounds that were either in tune (congruent condition) or had a mistuned component (incongruent condition). The authors found that the incongruent condition elicited an enlarged object-related negativity (ORN) in participants with hearing impairments. The ORN is an event-related potential (ERP) component that reflects the perception of a mistuned low tonal element of a complex tone [e.g., (6–8)]. The authors suggested that HL increases neural excitability in auditory cortex which could be related to deficits in inhibitory control. Finally, in addition to inhibitory control, HL can also considerably influence general and emotional well-being in the elderly (9, 10).

Recent neuroimaging work showed that HL is also associated with a specific neuronal reorganization, most notably in networks responding to emotional stimuli (3). The authors reported that HL reduces the engagement of the limbic regions during processing of affective stimuli (e.g., including the left amygdala, left parahippocampus etc.), likely due to affected processing of acoustic features or valence. Furthermore, it was shown that negative sounds improve the functioning of “backward connections from the amygdala to the auditory cortex,” while the “forward connections from the auditory cortex to the amygdala” are modulated by the acoustic features of a sound (11). Therefore, it is likely that continuous loss of hearing acuity may affect the reported connectivity patterns during processing of emotional sounds and result in hindered perception or misclassification thereof (3).

The correct identification of non-verbal acoustic and facial affective cues is a vital component of adequate interpersonal communication (12). However, this process becomes particularly challenging when the emotional valence of different communication channels (auditory, visual) is incompatible, resulting in emotional conflict (13, 14). Processing of such conflict is costly as shown in slower responses, increased error rates and conflict- and emotion-specific ERP components (15–18).

For instance, Zinchenko et al. (19) ran EEG experiments where they presented participants (groups of older and younger adults) with multisensory dynamic stimuli: short video clips of actors facially expressing and vocalizing negative or neutral emotions. The incongruence was created between non-emotional vowel category (cognitive task of Experiment 1) and emotional valence of visual and audio dimensions (emotional task of Experiment 2). More specifically, in Experiment 1 participants were asked to identify the vowel (i.e., “A” or “O”) and ignore the emotional valence of stimuli, while in Experiment 2 the task was to report emotion of the voice (negative or neutral) regardless of matching or mismatching emotional and neutral facial expressions. Therefore, the authors varied the emotion of the target dimension (neutral, negative) and the nature of conflict was either emotional or cognitive.

As a result, negative emotions improved emotional conflict processing in younger (18) and older adults (19). In more detail, the conflict effect (i.e., RT difference between incongruent and congruent conditions) was smaller in the negative emotion condition relative to the neutral condition. Similarly, negative emotion was also shown to modulate conflicts that arise between opposing non-emotional stimulus dimensions [i.e., *cognitive conflicts;* (19); see also (20, 21), for similar findings]. Besides behavioral modulation of cognitive and emotional conflicts, negative emotions also resulted in conflict-specific ERP responses (18, 19).

Specifically, younger adults showed a conflict specific dissociation of the N100 during processing of cognitive and emotional conflicts (18). The N100 is a negative-going wave that peaks 80–120 ms after sound onset and was most often found over the fronto-central region of the scalp [see (22) for auditory N100]. This component is modulated by attention (23), emotion (24), and congruence (25). In the cognitive conflict task, the conflict effect was observed to be larger for negative relative to neutral trials, while in the emotional conflict task the conflict effect was more pronounced in neutral as compared to negative trials (18). Another component sensitive to conflict processing is the P200 (positive wave that peaks around 200 ms post-stimulus). The P200 increases for emotional compared to neutral stimuli (26, 27), and its amplitude decreases for incongruent stimuli in both cognitive and emotional conflict tasks (18, 28). Lastly, a well-described neural marker of conflict processing is the N200 (i.e., negative-going deflection that peaks 200–350 ms post-stimulus) that elicits larger amplitude in response to incongruent than congruent stimuli (29–31). The N200 conflict effect was observed at fronto-central (20, 30, 32) and posterior electrode-sites (18, 33).

Although the role of HL in various cognitive functions has been studies extensively in the last years (34–36), relatively little is known how decreasing hearing capacity affects the role of emotions in cognitive and emotional conflict processing. The detection of conflict in processing of emotional information is vital in successful interpersonal communication and social adaptation. Therefore, it is possible that social isolation observed in HL older adults (37) may at least be partially related to problems in the processing of complex social information that may contain emotional conflict. In order to test this hypothesis, we used multisensory dynamic stimuli and manipulated them in a way to make emotion either task-irrelevant (the conflict was created between non-emotional stimulus dimensions; cognitive conflict) or task-relevant, where emotional stimulus dimensions were made incongruent [emotional conflict; see (18)]. Specifically, we explored whether the cognitive and emotional conflicts influence early perceptual processes [e.g., N100, P200; (18)] and whether negative emotion is able to modulate the two types of conflict [N200; (20, 38)] in participants with varying degrees of hearing capacity.

Based on previous findings that HL results in a reduction of gray matter volume in frontal cortex and particularly control-specific anterior cingulate cortex (ACC), we expected that the HL group would result in particularly hindered performance in both cognitive conflict task (39, 40) and in the emotional conflict task (19). Additionally, it was expected that negative targets would improve performance in both cognitive and emotional tasks in the NH group (19). On the other hand, as several previous studies indicated reduced capabilities to process emotional information related to moderate HL [e.g., (3)], we hypothesized that emotional targets would have no influence on both types of conflict in HL older adults. Due to its precise temporal resolution and millisecond precision, measuring EEG further allowed testing at what stage does HL influence processing of affective information. Finally, multisensory emotional and cognitive information optimizes behavioral responses in older adults (41–45). Therefore, we used dynamic multisensory emotional and neutral video stimuli in order to elicit the most optimal behavioral and neural responses [e.g., (46, 47)].

In line with our recent findings, we expected that incongruent cognitive and emotional tasks would result in a bigger N100 amplitude increase for negative than for neutral targets in older adults with NH (19). Additionally, we predicted that incongruent relative to congruent trials will result in a smaller P200 response in the two types of conflict (18, 28). These conflict-specific N100 and P200 responses were previously found at either anterior (28, 48, 49) or posterior electrode-sites (48, 50) in younger adults and at anterior electrode-sites only in older adults (19).

We also expected that incongruent stimuli would elicit a larger N200 responses in both groups (18, 51). We hypothesized that emotional targets would not modulate the N200 in the two conflict types, since the modulation of this component seems to be observed for static unimodal pictures (20) but not for dynamic multisensory videos (18, 19).

## Methods

### Participants

Twenty-one NH older adults (see Table 1 for demographic information) and 21 older adults with HL with normal or corrected-to-normal vision participated in Experiment 1 and Experiment 2. The order of the two Experiments was counterbalanced and we kept at least 7 days in between the two testing days. All participants were right-handed (Edinburgh Handedness Inventory score ME = 89.6, *SD* = 11.7). Sample size was determined on the basis of previous studies that used identical paradigm and stimuli [e.g., (18, 19)]. On the basis of effect size measures provided in these studies, we determined that our sample size would be appropriate to detect an f(U) effect size of 0.33 with 85% power (partial eta2 = 0.1, groups = 2, number of measurements = 4), given an alpha level of 0.05 and a nonsphericity correction of 1. Participants had no history of alcoholism, drug abuse, neurological or psychiatric disorders as assessed via Structured Clinical Interview in DSM-IV [SCID-I; (52)] at the Day Clinic for Cognitive Neurology, University of Leipzig. Additionally, we used an instant dipstick drug test (Drogentest Multi-8/2-DT, Diagnostik Nord) to examine a possible use of eight drugs (amphetamine, buprenorphine, benzodiazepines, cocaine, methamphetamine, morphine/opiates, methadone, and cannabis) in both hearing groups. Older adults in the two groups did not differ in mean age [*t*_(40)_ = −1.76, *p* > 0.05] or mean years of education: HL group (all 11.6 years, *SD* = 2), NH group [mean = 11.2 years, *SD* = 1.46, *t*_(40)_ = −1.18, *p* > 0.2]. The two hearing groups came from the “Leipzig Cohort for Mind-Body-Emotion Interactions” (LEMON) database.

**Table 1:** Subject demographics and clinical characteristics.

	**Hearing loss (HL)**	**Normal hearing (NH)**	**Significance**
Participants	21	21	
Age	70.5	68.4	
Effortful control	18.0	18.5	
Depression	7.0	10.0	
Anxiety	5.5	8.5	
Stress	10.3	13.6	
**dB TRESHOLDS:**		
R 250	23.0	22.2	
R 500	25.0	19.4	^*^
R 1000	25.5	17.8	^**^
R 1500	27.5	18.0	^**^
R 2000	30.0	16.6	^***^
R 3000	33.3	22.2	^**^
R 4000	40.0	35.2	
R 6000	47.8	44.2	
R 8000	50.0	51.0	
L 250	24.0	23.8	
L 500	24.3	19.4	^*^
L 1000	25.5	16.2	^**^
L 1500	29.3	16.2	^***^
L 2000	31.3	18.2	^***^
L 3000	34.8	23.0	^**^
L 4000	40.3	34.8	
L 6000	51.8	45.4	
L 8000	53.5	47.8	

The table contains hearing thresholds (in dB) for frequencies between 250 and 8,000 Hz for the right and left ears.

* p < 0.05;

** p < 0.01;

*** *p < 0.001*.

Participants were screened with a pure-tone audiometric testing. As a result, older adults in the NH group showed thresholds equal to or lower than 30 dB in both ears at [all] frequencies crucial for speech perception [500–4,000 Hz, (53)]. Participants in the HL group had thresholds between 30 and 70 dB in [at least one of] the corresponding frequencies, which corresponded to mild to moderately-severe HL. Table 1 reports average hearing information at frequencies of interest for both groups. The HL participants did not rely on hearing aids.

Additionally and in line with previous literature (54), participants completed the Adult Temperament Questionnaire [effortful control subscale, ATQ; (55)] and Depression Anxiety Stress Scale [DASS; (56)]. Both groups had comparable results for effortful control, stress, anxiety, and depression (see Table 1 for details).

Participants were asked to rate expressiveness, arousal, and *emotion identification* of the complete videos, video streams alone, and audio streams alone [see Table 2 and stimulus material below for details; (57)]. The groups did not differ with regard to perceived expressiveness and arousal of the stimuli. Furthermore, the NH group rated the emotional material as more emotional compared to the neutral material. On the other hand, the HL group rated emotional voices as neutral and emotional faces as even more negative relative to neutral stimuli and relative to the NH group (see Supplementary Material for details). A written informed consent form was obtained from all participants and they were paid ~30 € for participation. The experiment was conducted in accordance with the principles of the Declaration of Helsinki and was also approved by the Ethics Committee of the University of Leipzig.

**Table 2:** Results of the video rating.

**Stimuli**		**Arousal**	**Expressiveness**	**Valence**
**HEARING LOSS GROUP**				
Complete video	Neutral	5.18 (3.21)	5.41 (2.78)	5.02 (0.24)
	Negative	4.62 (2.40)	4.63 (2.86)	1.31 (0.48)
Audio stream	Neutral	4.63 (1.32)	4.44 (1.43)	4.78 (0.25)
	Negative	5.10 (1.26)	4.94 (1.28)	4.16 (1.59)
Videos stream	Neutral	5.36 (2.98)	5.31 (3.07)	4.97 (0.31)
	Negative	5.02 (2.19)	4.82 (2.56)	1.91 (1.40)
**NORMAL HEARING GROUP**				
Complete video	Neutral	5.13 (2.84)	5.35 (2.14)	5.01 (0.20)
	Negative	4.94 (2.71)	4.76 (2.23)	1.75 (0.58)
Audio stream	Neutral	4.72 (1.13)	4.12 (1.08)	5.01 (0.23)
	Negative	4.81 (0.98)	4.43 (1.05)	1.65 (0.71)
Videos stream	Neutral	5.05 (1.43)	4.63 (1.46)	4.95 (0.42)
	Negative	4.88 (1.36)	4.68 (0.88)	1.82 (0.69)

### Stimulus material

**Figure 1:**
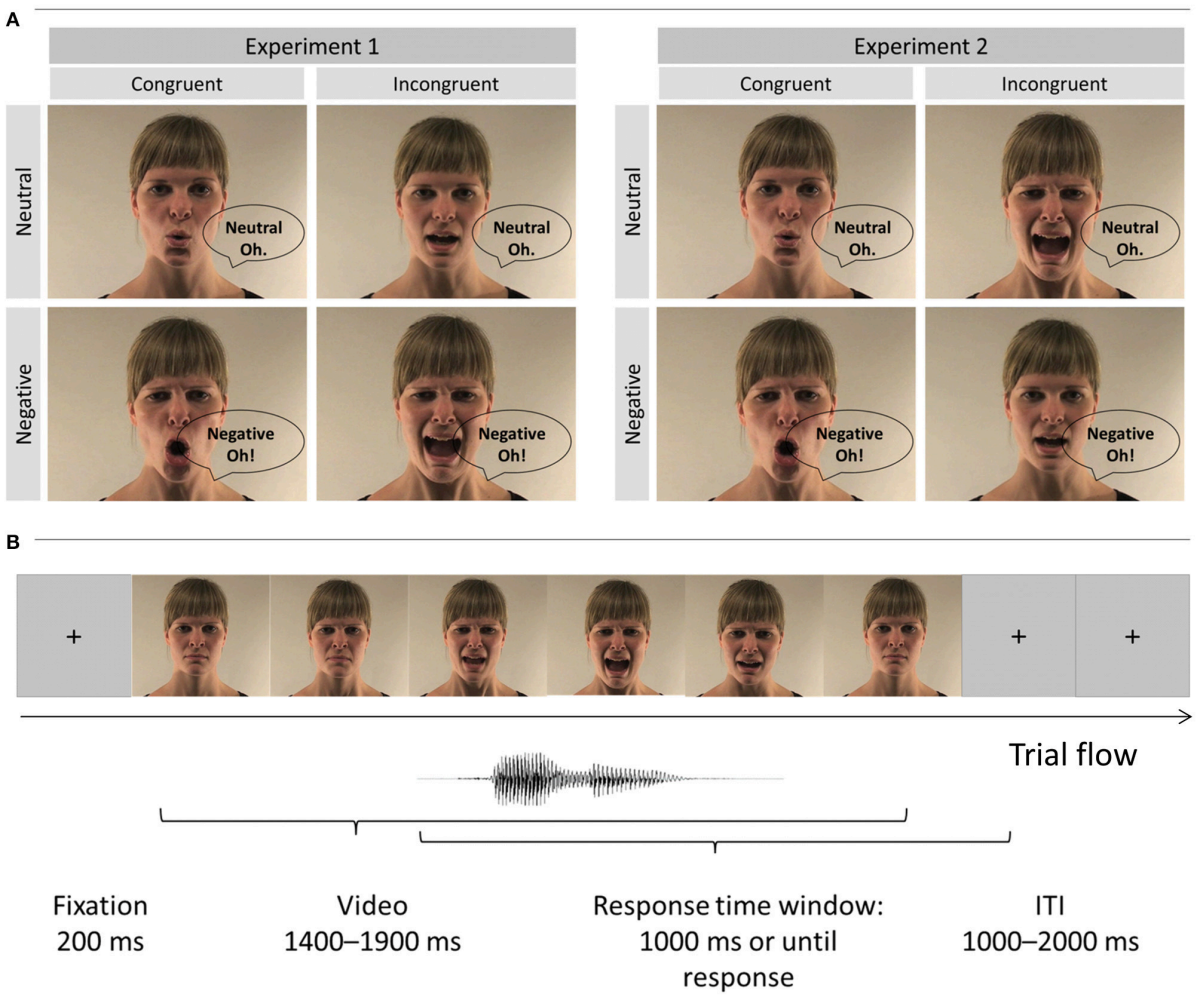
**(A)** Multimodal video stimuli of Experiments 1 and 2: Example of the female actor (publication of these images was approved by the actor) vocalized the interjections “A” and “O” in either a negative or neutral tone of voice. Incongruence was created by a mismatch of the vocalizations and the video components in Experiment 1 and mismatches in emotion of audio and video components in Experiment 2. **(B)** Example of a trial sequence. The trial started with a 200 ms fixation point that was followed by a video clip played in full length. The length of videos was varied (see Tables 3, 4 for details). The response time window was activated with the voice onset and lasted until response or a maximum of 1,000 ms. We also introduced a random inter-trial interval between 1,000 and 2,000 ms. Please note that speech bubbles “neutral” and “negative” refer to the [audio] target dimension (not visual dimension).

We validated experimental design, procedure and stimuli of the current study in our previous work (18, 19). Short video clips depicted either a male or a female actor articulating the vowel “A” and “O” in a neutral and negative (i.e., angry) tone of voice (see Figure 1A). The sounds in all videos were normalized to 70 dB by means of root mean square using Final Cut Pro 7 (Apple Inc.). In *Experiment 1*, we used these original videos to create 8 congruent and 8 incongruent stimuli by matching or mismatching vocalizations of the face and voice (e.g., voice pronouncing “A” with facial lip movement corresponding to “A” vs. “O,” respectively). Participants were asked to report the vowel pronounced by the voice (“A,” “O”). The onset of the original video sound was used for the overlay with the mismatching sound. In *Experiment 2*, we modified videos used in Experiment 1 and created 12 congruent and 12 incongruent emotional conflict videos. For this purpose, we mismatched the emotional valence of the face and voice (e.g., face [lip movements] pronouncing a neutral “A” and the corresponding audio “A” that is pronounced emotionally, Figure 1). Again, the onset of the original video sound was used for the precise overlay of the incongruent voice with the facial expression and lip movement in both incongruent conditions (negative [neutral] face—neutral [emotional] voice). Note that in Experiment 2 we always matched the vocalization of the face and voice. The task in this experiment was to report the emotional valence of the voice (negative, neutral). Thus, Experiments 1 and 2 were very similar, but differed in the task instruction and the combination of audio and visual stimuli. Additionally, the video duration in all conditions varied from 1 to 2 s (see Table 4). All conditions in Experiments 1 and 2 were comparable in time before the audio onset and total video durations (see Table 3 and Supplementary Material for details).

**Table 3:** Timing of video stimuli of Experiment 1.

**Video condition (the “vowel” specifies the interjection)**	**Time before start of the movement (ms)**	**Time before start of the audio sound (ms)**	**Total video duration (ms)**
**FEMALE**			
Neutral congruent “A”	240	561	1,400
Neutral congruent “O”	240	740	1,480
Negative congruent “A”	240	665	1,880
Negative congruent “O”	240	846	1,840
Face Neutral “A”—Voice Neutral “O”	240	540	1,400
Face Neutral “O”—Voice Neutral “A”	240	562	1,480
Face Negative “A”—Voice Negative “O”	240	680	1,880
Face Negative “O”—Voice Negative “A”	240	630	1,840
**MALE**			
Neutral congruent “A”	240	475	1,400
Neutral congruent “O”	240	560	1,400
Negative congruent “A”	240	450	1,400
Negative congruent “O”	240	540	1,400
Face Neutral “A”—Voice Neutral “O”	240	520	1,400
Face Neutral “O”—Voice Neutral “A”	240	635	1,400
Face Negative “A”—Voice Negative “O”	240	580	1,400
Face Negative “O”—Voice Negative “A”	240	490	1,400

**Table 4:** Timing of video stimuli of Experiment 2.

**Video condition (the “vowel” specifies the interjection)**	**Time before start of the movement (ms)**	**Time before start of the audio stream (ms)**	**Total video duration (ms)**
**FEMALE**			
Neutral congruent “A”	240	561	1,400
Neutral congruent “O”	240	740	1,480
Negative congruent “A”	240	665	1,880
Negative congruent “O”	240	846	1,840
Face Neutral—Voice Negative “A”	240	590	1,400
Face Neutral—Voice Negative “O”	240	860	1,480
Face Negative—Voice Neutral “A”	240	683	1,880
Face Negative—Voice Neutral “O”	240	659	1,840
**MALE**			
Neutral congruent “A”	240	475	1,400
Neutral congruent “O”	240	560	1,400
Negative congruent “A”	240	450	1,400
Negative congruent “O”	240	540	1,400
Face Neutral—Voice Negative “A”	240	328	1,400
Face Neutral—Voice Negative “O”	240	500	1,400
Face Negative—Voice Neutral “A”	240	590	1,400
Face Negative—Voice Neutral “O”	240	600	1,400

We observed no differences between conditions with regard to emotion identification, expressiveness and arousal (see Supplementary Material for details). We also tested whether videos differed with regards to movement. For this purpose, we quantified per-pixel changes in light intensity (luminance) between video frames (58). Subsequently, we used a Kruskal–Wallis test to compare the two emotion and two vowel conditions. As a result, there were no differences except for negative relative to neutral stimuli showing a higher number of movements (X^2^ = 5.33, *p* < 0.05). Since angry expressions are naturally more dynamic and intense [e.g., (59)] the observed difference is expected in naturalistic stimuli. Nevertheless, these motion differences should have no effect on final results, since we focused on the *interaction* of congruence and emotion. Finally, we found no motion differences between different vowels (X^2^ = 1.25, *p* > 0.2).

Both Experiment 1 and Experiment 2 consisted of four blocks with 52 videos in each block (negative = 26 videos, incongruence = 50%) that were pseudo-randomized and administered in a 2 (emotional, neutral) by 2 (congruent, incongruent) factorial design.

### Procedure

Both Experiments were performed in a sound-attenuated booth. Participants were seated about 1 m from a computer screen and audio stimuli were delivered via headphones. After 200 ms fixation cross participants watched videos stimuli in full duration (i.e., response did not terminate video presentation; see Figure 1B). In Experiment 1, the task was to identify vocalization of voices (either “A” or “O”), while emotional valence of the face and voice were (i) task-irrelevant and (ii) always matched. In Experiment 2, the task was to report the emotional valence of the voice (negative, neutral). We also introduced probe trials (10% of all trials presented randomly throughout experiment) when participants were additionally asked to report the vocalization of the face (i.e., lip movement, i.e., “A” or “O” in Experiment 1; emotion of the face in Experiment 2). This was done to ensure that faces were not ignored. These questions were not limited in time, and were not included into further analyses (all participants answered >90% questions correctly in both Experiments). Main questions had a response time-window of 1000 ms and started from voice onset. Participants saw a “try to respond faster” sign for 200 ms in case if they did not respond within the given time-window. In case of an incorrect response the word “incorrect” appeared on the screen. We counterbalanced button presses across participants and introduced a random intertrial duration between 1,000 and 2,000 ms. Lastly, in order to make sure that participants understood the task requirement we asked them to write the instructions down on a sheet of paper. All participants were able to correctly describe the task.

### EEG recording and pre-processing

We used Brain Vision Recorder (Brain Products GmbH, Munich, Germany) to record data from 59 Ag/AgCl electrodes (10-10 system) at a sampling rate of 500 Hz. The reference was at left mastoid, and ground was at the sternum. We measured vertical and horizontal electro-oculogram to reject artifacts and kept impedance level below 5 kΩ.

For the EEG data analyses we used the FieldTrip (v0.20120501) toolbox (60) running on Matlab 8.1 R2013a (The Mathworks, Natrick, USA). After re-referencing electrodes offline to linked mastoids we split the data into longer epochs (±2,000 ms time-locked to the voice onset) and rejected those epochs that contained excessive muscle activity or jump artifacts. We then band-pass filtered the data using a two-pass Butterworth IIR filter with a frequency pass-band of 0.1–100 Hz (order of four).We also applied principal components analysis after preprocessing, thus reducing dimensionality of the data and preserving α = 0.99 of the variance (61). A *fastica* algorithm was used for the independent component analysis (ICA). In the following step we have rejected components that showed ocular, muscle, heart, and electrode artifacts (number of components removed in Experiment 1: mean = 12, *SD* = 3, ~16% of trials; in Experiment 2: mean = 14, *SD* = 4.1, ~15% of trials). Finally, we have visually inspected individual epochs and discarded those epochs that contained artifacts.

### Data analysis

Smaller epochs time-locked to the voice onset (−200 to 1,000 ms) were selected for the statistical analysis. First, we band-pass filtered continuous EEG data using a two-pass Butterworth IIR filter with a frequency pass-band of 0.5–30 Hz, and then calculated averaged activity for each participant and for each session and condition after applying a 200 ms baseline correction before the voice onset (18, 19). Furthermore, in line with previous literature (18, 20, 48), four regions of interest (ROIs) were defined: left anterior (FP1, AF3, AF7, F3, F5, F7, FC3, FC5, FT7), right anterior (FP2, AF4, AF8, F4, F6, F8, FC4, FC6, FT8), left posterior (CP3, CP5, TP7, P3, P5, P7, PO3, PO7, O1), and right posterior (CP4, CP6, TP8, P4, P6, P8, PO4, PO8, O2). The following time-windows were used to identify peak latencies separately for each participant and each condition: 70–110 ms (N100), 140–225 ms (P200), and 240–380 ms (N200) as suggested by Luck and Kappenman (62). For a mean amplitude analysis we used averaged activity that fell within 40 ms (i.e., 20 ms before and after) of individual peaks from the group mean ERPs. Subsequently a repeated-measures ANOVA was calculated for each time-window, using emotion (emotional, neutral), congruence (congruent, incongruent), region (anterior, posterior), and side (left, right) as within-subject factors and group (NH, HL) as a between-subject factor. In the results section, we report statistically significant effects that involved the critical factors emotion, congruence, and group.

## Results

### Experiment 1

#### Behavioral data

##### RT data

**Figure 2:**
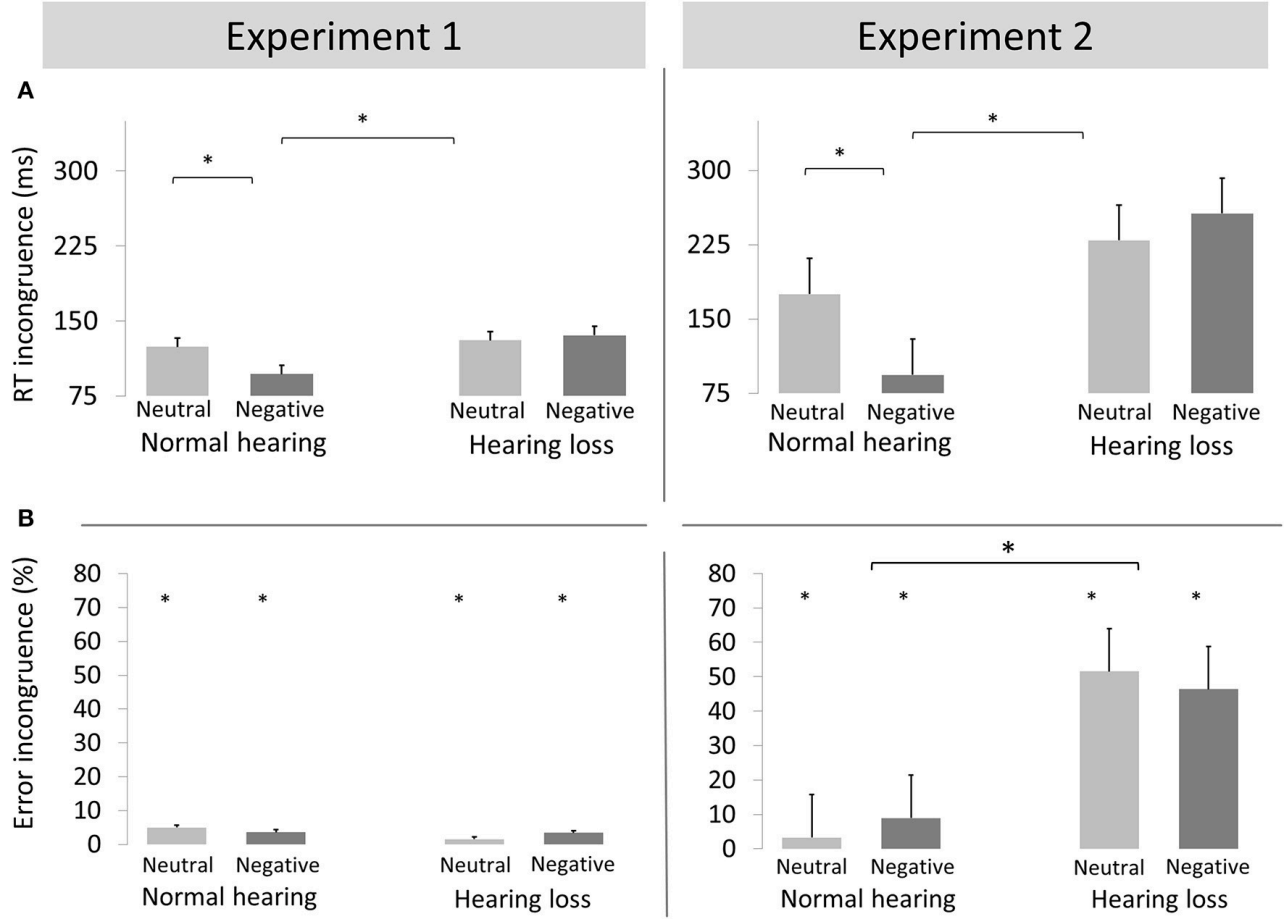
Reaction times **(A)** and Error rates **(B)** (mean + standard error of the mean) incongruence effect (incongruent minus congruent) for neutral and negative conditions of Experiment 1 (left) and Experiment 2 (right) for adults with normal hearing and with hearing loss. ^*^p < 0.05.

We report an interaction of emotion, congruence and group [*F*_(1, 40)_ = 6.89, *p* < 0.02, η_*p*_^2^ = 0.147; see Figure 2]. *Post-hoc* analyses by group revealed an interaction of emotion and congruence in the NH group [*F*_(1, 20)_ = 10.36, *p* = 0.004, η_*p*_^2^ = 0.341] but not in the HL group [*F*_(1, 20)_ = 0.323, p > 0.5, η_*p*_^2^ = 0.016]. In the NH group, the conflict effect was smaller for negative emotion targets [97 ms; *F*_(1, 20)_ = 32.18, *p* = 0.01, η_*p*_^2^ = 0.617] than neutral targets p124 ms; *F*_(1, 40)_ = 64.119, *p* < 0.01, η_*p*_^2^ = 0.762].

##### Errors

Incongruent stimuli elicited more errors than congruent stimuli [F_(1, 40)_ = 6.54, *p* < 0.02, η_*p*_^2^ = 0.150]. No other main effects or interactions reached significance (all p's > 0.05).

#### ERP data

##### N100 range

**Figure 3:**
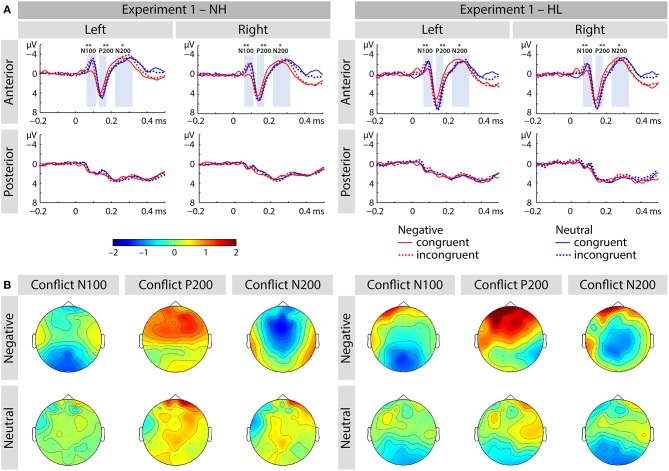
**(A)** Averaged ERP waveforms at selected electrodes [left anterior (FP1, AF3, AF7, F3, F5, F7, FC3, FC5, FT7), right anterior (FP2, AF4, AF8, F4, F6, F8, FC4, FC6, FT8), left posterior (CP3, CP5, TP7, P3, P5, P7, PO3, PO7, O1), and right posterior (CP4, CP6, TP8, P4, P6, P8, PO4, PO8, O2)] showing responses to congruent and incongruent, emotional and neutral conditions of Experiment 1. **(B)** Topographic distribution depicting conflict effect as amplitude differences (incongruent – congruent) for each of the corresponding ERP components (i.e., N100, P200, and N200). The asterisks represent statistical significance: ^*^p < 0.05, ^**^p < 0.01.

**Figure 4:**
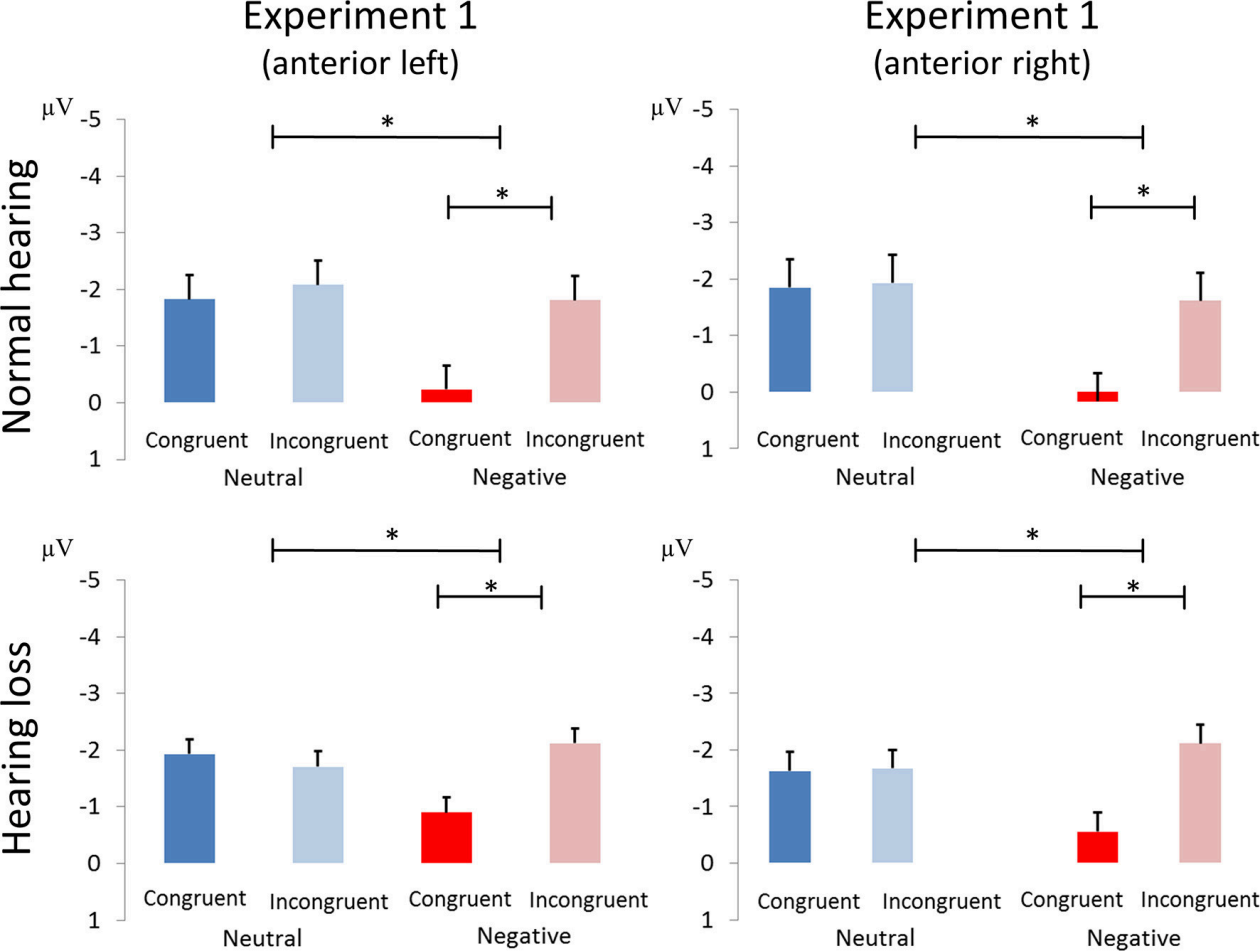
Amplitude differences between congruent and incongruent neutral and negative emotion stimuli in the N100 of Experiment 1. The asterisks represent statistical significance: ^*^0.01.

We found main effects of emotion [*F*_(1, 40)_ = 5.38, *p* < 0.03, η_*p*_^2^ = 0.119; see Figure 3] and congruence [*F*_(1, 40)_ = 13.04, *p* < 0.001, η_*p*_^2^ = 0.246], as well as an interaction of emotion and congruence [*F*_(1, 40)_ = 9.54, *p* < 0.01, η_*p*_^2^ = 0.193]. *Post-hoc* analyses revealed larger mean congruence effect for negative [*F*_(1, 40)_ = 19.36, *p* < 0.001, η_*p*_^2^ = 0.326] but not for neutral stimuli [*F*_(1, 40)_ = 0.116, *p* > 0.7, η_*p*_^2^ = 0.003]. The interaction between emotion, congruency, and group was not significant (see Figure 4).

##### P200 range

We report an interaction of region and congruence [*F*_(1, 40)_ = 7.00, *p* < 0.02, η_*p*_^2^ = 0.149]. Incongruent stimuli elicited an increased amplitude over the anterior electrode-sites [*F*_(1, 40)_ = 5.56, *p* < 0.03, η_*p*_^2^ = 0.122], but not over posterior sites [*F*_(1, 40)_ = 0.127, *p* > 0.7, η_*p*_^2^ = 0.03].

##### N200 range

We found no significant main effects or interactions in the N200 time range.

To summarize, in Experiment 1 we tested whether *task-irrelevant* emotion influences cognitive conflict processing in two elderly groups of participants with different hearing capacities. As a result, emotion facilitated behavioral conflict processing by reducing the conflict effect in the NH but not in the HL group. However, negative emotion modulates cognitive conflict in the N100 of both hearing groups, putatively indicating that emotion modulates early conflict-specific processing in spite of HL. Interestingly, both groups showed a control-specific P200 conflict effect only at anterior electrode-sites. Finally, we did not find a N200 conflict effect in either one of the groups. In Experiment 2 we further tested whether HL modulates the role emotional valence of the target in the emotional conflict task.

### Experiment 2

#### Behavioral data^1^

##### RT data

We observed an interaction of emotion, congruence, and group [*F*_(1, 40)_ = 10.78, *p* < 0.01, η_*p*_^2^ = 0.212; see Figure 2]. *Post-hoc* by group revealed an interaction of emotion and congruence in the NH group [*F*_(1, 20)_ = 15.03, *p* < 0.01, η_*p*_^2^ = 0.429] but not in the HL group [*F*_(1, 20)_ = 1.13, *p* > 0.3, η_*p*_^2^ = 0.054]. In the NH group, the conflict effect was smaller for negative [93 ms; *F*_(1, 20)_ = 17.53, *p* < 0.01, η_*p*_^2^ = 0.467] than neutral trials [175 ms; *F*_(1, 20)_ = 128.22, *p* < 0.001, η_*p*_^2^ = 0.865].

##### Errors

We found main effect of congruence [*F*_(1, 40)_ = 116.03, *p* < 0.001, η_*p*_^2^ = 0.730] and an interaction of congruence and group [*F*_(1, 40)_ = 67.15, *p* < 0.001, η_*p*_^2^ = 0.610]. *Post-hoc* analyses revealed that the conflict effect was larger in the HL group [46.6%, *F*_(1, 20)_ = 86.08, *p* < 0.001, η_*p*_^2^ = 0.811] than the NH group [6.7%, *F*_(1, 20)_ = 21.42, *p* < 0.001, η_*p*_^2^ = 0.482].

##### N100

**Figure 5:**
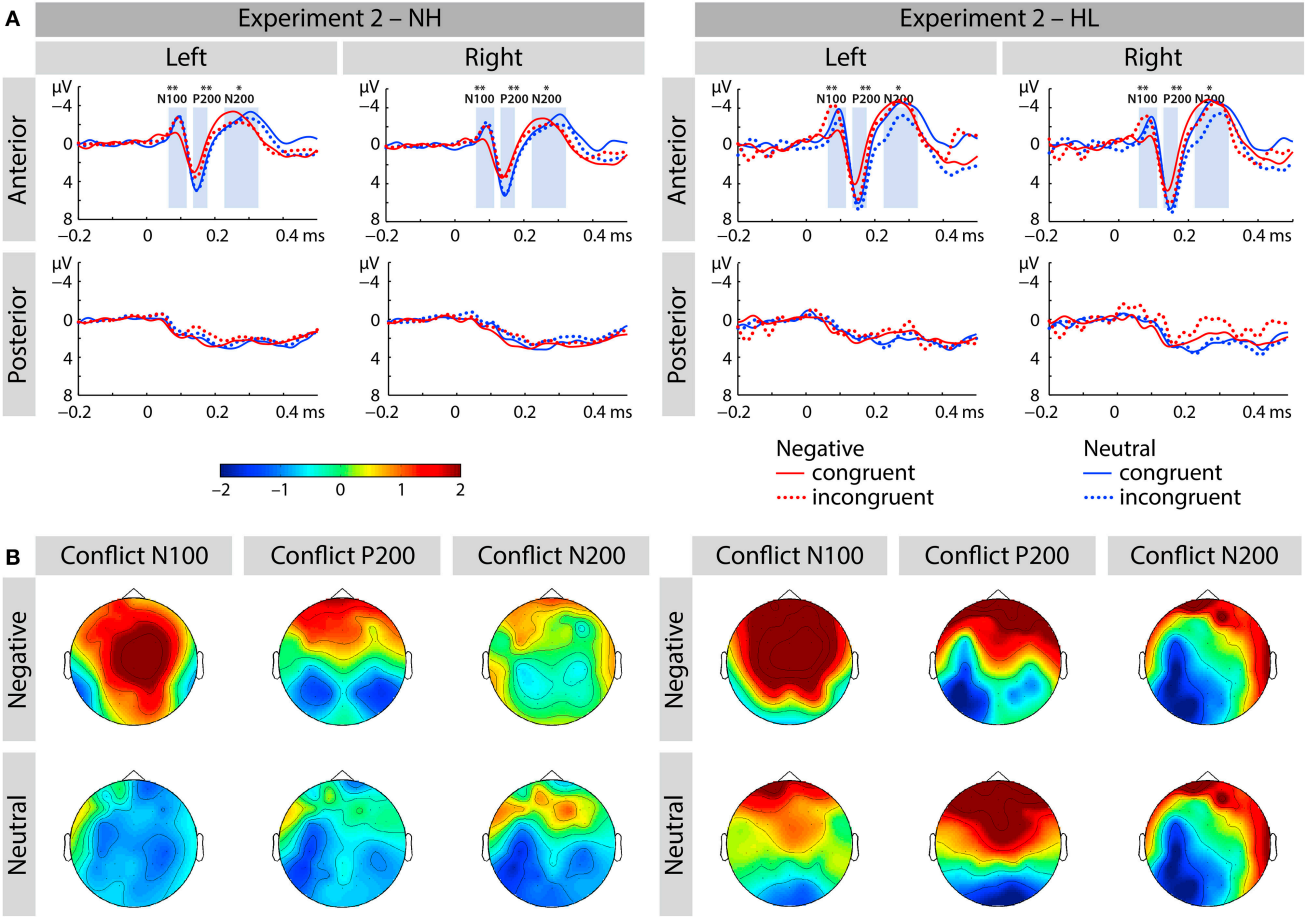
**(A)** Averaged ERP waveforms at selected electrodes [left anterior (FP1, AF3, AF7, F3, F5, F7, FC3, FC5, FT7), right anterior (FP2, AF4, AF8, F4, F6, F8, FC4, FC6, FT8), left posterior (CP3, CP5, TP7, P3, P5, P7, PO3, PO7, O1), and right posterior (CP4, CP6, TP8, P4, P6, P8, PO4, PO8, O2)] showing responses to congruent and incongruent, emotional and neutral conditions of Experiment 2. **(B)** Topographic distribution depicting conflict effect as amplitude differences (incongruent – congruent) for each of the corresponding ERP components (i.e., N100, P200, and N200). The asterisks represent statistical significance: ^*^p < 0.05, ^**^p < 0.01.

**Figure 6:**
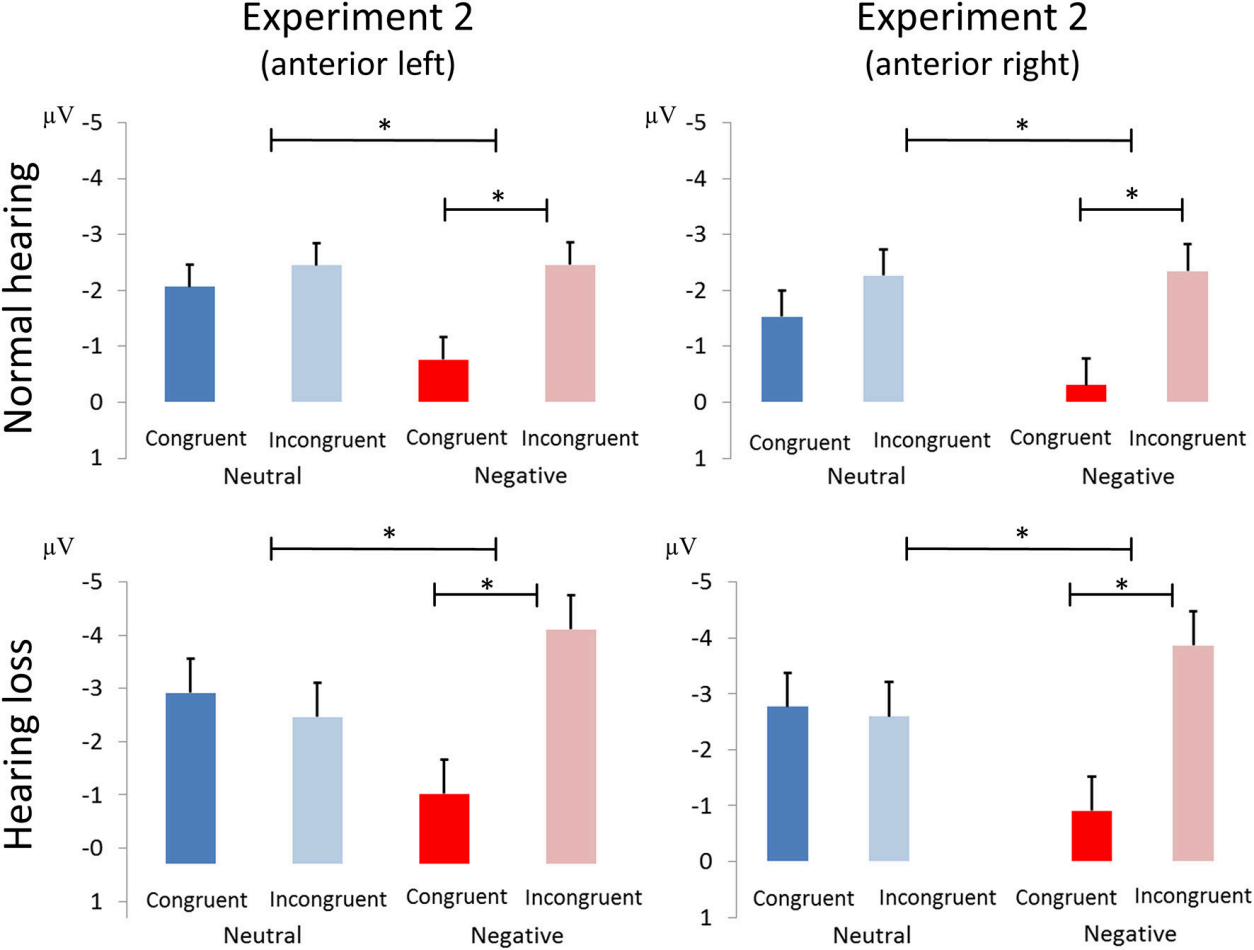
Amplitude differences between congruent and incongruent neutral and negative emotion stimuli in the N100 of Experiment 2. The asterisks represent statistical significance: ^*^0.01.

We report an interaction of emotion and congruence [*F*_(1, 40)_ = 13.68, *p* < 0.01, η_*p*_^2^ = 0.255; see Figure 5]. Incongruent stimuli elicited larger N100 amplitudes than congruent stimuli in the negative emotion condition [*F*_(1, 41)_ = 17.69, *p* < 0.001, η_*p*_^2^ = 0.301] but not in the neutral condition [*F*_(1, 41)_ = 1.21, *p* > 0.25, η_*p*_^2^ = 0.029]. We observed no main effect or interactions with the factor group (all p's > 0.05; see Figure 6).

##### P200

The main effect of emotion was significant [*F*_(1, 40)_ = 11.25, *p* < 0.01, η_*p*_^2^ = 0.220]. The interaction of emotion and region was also significant [*F*_(1, 40)_ = 9.2, *p* < 0.01, η_*p*_^2^ = 0.187]. We observed that negative stimuli elicited smaller P200 responses than neutral stimuli and this effect was larger over the anterior brain region [*F*_(1, 41)_ = 13.22, *p* < 0.01, η_*p*_^2^ = 0.244] relative to posterior sites [*F*_(1, 41)_ = 4.39, *p* = 0.042, η_*p*_^2^ = 0.097].

##### N200

We observed an interaction of emotion and group [*F*_(1, 40)_ = 4.23, *p* < 0.05, η_*p*_^2^ = 0.096]. Negative stimuli elicited marginally larger N200 responses in the HL group [*F*_(1, 20)_ = 4.28, *p* = 0.052, η_*p*_^2^ = 0.176], but not in the NL group [*F*_(1, 20)_ = 0.340, *p* > 0.5, η_*p*_^2^ = 0.017]. We also found an interaction of region and congruence [*F*_(1, 40)_ = 14.28, *p* < 0.01, η_*p*_^2^ = 0.263]. *Post-hoc* analyses revealed that incongruent relative to congruent stimuli led to smaller N200 responses at anterior electrode-sites [*F*_(1, 41)_ = 4.2, *p* < 0.5, η_*p*_^2^ = 0.097], but not at posterior ones [*F*_(1, 41)_ = 2.064, *p* > 0.15, η_*p*_^2^ = 0.048].

In summary, Experiment 2 tested how HL modulates the role of emotion of the target in emotional conflict processing. As expected, we found that negative stimuli improved processing of emotional conflict by reducing the RT conflict effect in the NH group but not in the HL group. The N100 response showed an age-independent interaction of emotion and control: incongruent negative as compared to neutral stimuli resulted in larger N100 responses than congruent stimuli. We also found that the effect of emotion differed across the regions in the P200 of both groups, and it also varied between the two hearing groups in the N200.

##### Omnibus ANOVA

In the omnibus ANOVA we directly compared the results of Experiment 1 and Experiment 2. For each time window, a repeated-measures ANOVA was calculated using conflict type (cognitive, emotional), emotion (emotional, neutral), congruence (congruent, incongruent), region (anterior, posterior), and side (left, right) as within-subject factors and hearing group (normal hearing, hearing loss) as a between-group factor.

### Results

#### Behavioral data

##### RT data

We found a 4-way interaction of experiment x congruence x emotion x group [*F*_(1, 40)_ = 4.84, *p* = 0.034, η_*p*_^2^ = 0.108]. Figure 2 shows that the benefit of negative emotion was more pronounced in the emotional than the cognitive conflict task. Nevertheless, the pattern of results was identical for both experiments: the conflict effect was reduced for emotional compared to neutral conflicts in the NH group but not in the HL group.

##### Error

We found an interaction of conflict type, congruence, and group [*F*_(1, 40)_ = 46.09, *p* < 0.001, η_*p*_^2^ = 0.535]. Incongruent relative to congruent trials resulted in increased errors in Experiment 1 [*F*_(1, 40)_ = 6.54, *p* < 0.02, η_*p*_^2^ = 0.150]. In Experiment 2, the conflict effect was larger in the HL group [*F*_(1, 20)_ = 86.08, *p* < 0.001, η_*p*_^2^ = 0.811] than the NH group [*F*_(1, 20)_ = 21.42, *p* < 0.001, η_*p*_^2^ = 0.482].

##### N100

We observed an interaction of emotion x congruence [*F*_(1, 40)_ = 10.51, *p* < 0.01, η_*p*_^2^ = 0.208] as well as an interaction of region x congruence x emotion [*F*_(1, 40)_ = 6.41, *p* < 0.02, η_*p*_^2^ = 0.138]. Follow-up analysis over the two brain regions revealed an interaction of emotion and congruence in the anterior brain region [*F*_(1, 41)_ = 12.01, *p* < 0.01, η_*p*_^2^ = 0.226], but not in the posterior brain region [*F*_(1, 41)_ = 2.84, *p* > 0.05, η_*p*_^2^ = 0.065].

##### P200

We found a significant main effect of emotion [*F*_(1, 40)_ = 10.61, *p* < 0.01, η_*p*_^2^ = 0.210] as well as an interaction of region and emotion [*F*_(1, 40)_ = 15.98, *p* < 0.001, η_*p*_^2^ = 0.286]. Follow-up analyses showed that neutral stimuli generated larger amplitude than negative stimuli in the anterior brain region [*F*_(1, 41)_ = 14.78, *p* < 0.001, η_*p*_^2^ = 0.265], but not in the posterior brain region [*F*_(1, 41)_ = 2.19, *p* > 0.1, η_*p*_^2^ = 0.051]. We also observed an interaction of region and congruence. Incongruent stimuli produced enhanced amplitude in the anterior brain region, [*F*_(1, 41)_ = 9.14, *p* < 0.01, η_*p*_^2^ = 0.182], but not in the posterior region [*F*_(1, 41)_ = 1.49, *p* > 0.2, η_*p*_^2^ = 0.035].

Finally, we also found an interaction of congruence and hearing group [*F*_(1, 40)_ = 4.16, *p* = 0.048, η_*p*_^2^ = 0.094]. Incongruent relative to congruent stimuli elicited enhanced amplitude in the HL group [*F*_(1, 40)_ = 10.51, *p* = 0.002, η_*p*_^2^ = 0.208], but not in the NH group [*F*_(1, 20)_ = 5.215, *p* = 0.033, η_*p*_^2^ = 0.207].

##### N200

In the N200 we observed no main effect or interactions involving factors experiment, group, congruence, and emotion.

#### Discussion

The present set of experiments investigated the role of age-related HL on the influence of emotion on cognitive and emotional control with behavioral and ERP measures. In what follows, we examine in detail the results of the two conflict tasks and finally conclude with a general discussion.

##### Cognitive conflict

In this Experiment, participants were instructed to report the vowel expressed in videos (i.e., “A” or “O”) regardless of its emotional quality. As a result, both groups showed prolonged responses to incongruent compared to congruent stimuli as well as delayed responses to negative than to neutral stimuli. Most importantly, we observed that negative targets reduced the conflict effect in the normal hearing (NH) group, but not in the hearing loss (HL) group. Finally, emotion modulated the N100 conflict effect in both hearing groups, and incongruent stimuli elicited an increased P200 amplitude specifically over anterior electrode-sites in both groups.

First, we found that emotion does not benefit conflict processing in the HL group. Additionally, the participants' ratings of the stimuli showed that HL individuals rated negative targets as less emotional than participants of the NH group (Table 2). This is in accordance with what was reported by Husain et al. (3), who showed that moderate HL results in reduced brain activity in response to emotional targets and to structural changes in brain regions that are known to be involved in the processing of emotions. However, HL individuals rated negative *visual* stimuli as more negative than NH participants. This may imply that participants with HL relied more heavily on visual information when judging the emotional valence of stimuli and, as a form of overcompensation, rated negative visual stimuli as more negative.

In the EEG, we observed that emotional compared to neutral stimuli resulted in larger N100 conflict effect (18, 19) in both NH and HL adults. Negative emotion requires only some 100 ms to modulate early neural responses to incongruent stimuli in both hearing samples, potentially by increased or preferential allocation of attention to the target (18, 63). Due to high motivational relevance and saliency, visual and acoustic emotional stimuli attract attention (64, 65) and facilitate control processes (21, 66). Possibly, as emotional information in the visual domain was still available to the HL group and as these individuals seem to rely more heavily on visual information, the HL group could show an intact early neural response to the conflict, albeit without a corresponding behavioral facilitation. Alternatively, it is also possible that HL adults do actually process emotional characteristics of acoustic information to some degree during the early neural processing stages but not later on. In other words, this result implies that HL may reduce *confidence* for the perception of emotional tones with intact early neural responses to emotional auditory stimuli.

The P200 also resulted in a conflict effect over anterior electrode-sites in both hearing groups: incongruent P200 amplitude was smaller than congruent P200 amplitude. Increased attentional demands correlate with decreased P200 responses (28, 67). Consequently, the observed reduction in P200 to incongruent stimuli may be explained by distractor-related increase in attentional demands (15, 68).

Finally, we observed no conflict- or emotion-related main effects and interactions in the N200. This ERP component is evoked when prepotent responses have to be inhibited (31, 69). Therefore, the observed reduced effortful control and executive functions in older individuals may explain the absence of the N200 conflict effect in both hearing groups [see (70) for comparable findings; (19, 71)].

To summarize, the HL group show intact initial processing of negative auditory stimuli, but reduced confidence at later processing stages. Further, the current results indicate that despite problems with processing of emotional auditory information, HL participants were able to process other acoustic features of sounds (interjections “Ah” and “Oh”) as indicated by comparable overall conflict effects and error rates.

##### Emotional conflict

In this experiment, the task was to report the emotion of the auditory stimulus dimension regardless of the emotion of the visual facial expression, while vocalizations of the face (lip movement) and voice were always matched and task-irrelevant. As a result, emotion facilitated behavioral performance in NH participants, but not in the HL group. Moreover, HL individuals performed at chance level, with error rates ~50% in incongruent trials. In the EEG, we found a valence-specific N100 conflict response in the two groups: (i) the N100 amplitude was larger for incongruent relative to congruent stimuli, and (ii) this effect was greater for negative than neutral stimuli. Finally, in the P200 and N200 responses we also observed conflict- and valence-specific effects.

Behavioral RT conflict processing was improved for emotional stimuli in NH adults, while HL participants showed a chance performance in response to incongruent stimuli in the emotional conflict task. As HL was shown to diminish processing of acoustic emotional information in the current and previous studies (3, 11), processing of emotional conflict was especially problematic for HL adults. In other words, the HL group could have purely relied on the visual input due to the inability to make use of acoustic stimuli and, therefore, performed at chance level.

In the EEG, we found that emotional rather than neutral stimuli led to an increased N100 conflict effect: we found a larger N100 response to incongruent stimuli in the negative, but not in neutral trials. This effect was comparable in both hearing groups. Therefore, these results indicate that moderate HL does not diminish the processing of emotional cues *completely* as participants must have detected some emotional information in the acoustic signal that conflicted with the concurrent visual input.

Kumar et al. (11) showed that the backward connections from the amygdala to the auditory cortex were modulated by negative sounds. On the other hand, the acoustic features of a sound modulated the forward connections from the auditory cortex to the amygdala (11). These forward and backward projections are thought to function jointly to process acoustic stimuli (11). Husain et al. (3) hypothesized that hearing-loss related sound deprivation may lessen the available acoustic and/or valence information for the auditory cortex-amygdala interface. The authors propose that, people with HL may exhibit a dulled response to emotional stimuli as they may lack necessary acoustic or valence information required for an adequate emotional response. The current results demonstrate that processing of emotional stimuli is not delayed in HL participants, but these individuals tend to misclassify acoustic emotional information. Additionally, HL could have specifically impacted backward connections from the amygdala to the auditory cortex, thus letting some emotional information still reach the amygdala via the forward connections and to evoke emotion-specific early neural responses in the N100.

We also observed that negative stimuli elicited smaller P200 amplitude than neutral stimuli. Emotion-specific reduction in the P200 response may be driven by attentional capture by negative vocalizations (67), even in the HL group. As discussed above, this finding also implies that HL may result in reduced *confidence* in the perception of emotional sounds, while early neural responses to such stimuli remain intact.

In the N200 we observed that incongruent stimuli elicited larger responses than congruent stimuli over anterior, but not posterior electrodes. Previous findings suggest that the N200 is an index of conflict monitoring, with its amplitude varying as a function of attentional control required for conflict processing (31, 72, 69). Therefore, an increased N200 response may reflect increased executive demands to process the incongruent stimuli. Finally, negative stimuli elicited increased amplitudes in comparison to neutral stimuli in the HL group, but not in the NH group. We conclude that this may reflect additional demands, uncertainty and difficulty to process emotional stimuli in the HL participants as suggested by previous fMRI research (3).

#### General discussion and limitations

The current results replicate previous findings that negative emotion facilitates both cognitive and emotional conflict processing by reducing the RT conflict effect (18–20, 73). Emotional stimuli attract attention due to their motivational relevance for survival (64, 65) and trigger cognitive control processes (66). Processing of emotional stimuli is also known to enhance the readiness to act (74) and speed up executive control in both conflict types (16, 18).

Interestingly, no emotion-related behavioral facilitation was observed in the HL group. It was suggested that the age-related gradual increase in HL may promote social isolation (2, 37). As a consequence, the emotion processing limbic network may also be impacted as has been shown in aging and tinnitus research (75–77). Our results further indicate that social isolation in moderate HL may in part be caused by problems in processing emotional information. Although this topic has not been investigated in much detail in older individuals, it has been shown that children (of up to 9 years old) with mild-to-moderate HL are less able to understand complex social signals such as sarcasm, due to an inability to extract the sarcastic intonation from acoustic information (78). Additionally, Segal and Kishon-Rabin (79) showed that younger adults with mild HL may have problems with the comprehension of the stressed words in a sentence. Processing of emotional cues is even more challenging when they are complemented by emotional cues from different communication channels [audio, visual; (13, 14)]. As shown here, processing of such conflicts may become particularly burdensome for people with age-related HL.

Husain et al. (3) hypothesized that HL may diminish acoustic or valence cues required for the adequate processing of emotional information. Our results suggest that HL may not result in a general susceptibility to acoustic features that are available for processing as HL participants generally performed well in the cognitive conflict task. On the other hand, participants showed the strongest deprivation when the task required to identify the emotional valence of auditory targets. These findings are especially obvious since the multisensory stimuli in the two different conflict tasks were very similar.

Despite a lack of behavioral facilitation (Experiment 1) and chance level performance (Experiment 2), the HL group showed no difference in the emotion-modulated early (100 ms post-stimulus) conflict-specific responses. It is possible that the preserved processing of emotional information from the visual domain could facilitate early conflict specific neural processing in HL group. However, this explanation does not apply in the emotional conflict task, where visual emotion information was not available in the incongruent emotional condition (i.e., incongruent combination of a neutral face and a negative voice). These results may imply that HL in the selected frequencies does not completely restrict the processing of emotional cues from the acoustic signal. It appears that HL older adults were still able to process emotional cues to a certain degree; however, this did not result in any behavioral benefits, probably due to reduced confidence in the processing of emotional cue. This hypothesis is in line with our rating results, as well as with previous findings of Picou (80), who showed that HL participants exhibited a reduced range of emotional ratings.

The current study tested whether age-related moderate HL impacts how negative emotions impact cognitive and emotional control. However, it remains open whether we would observe the same result for positive emotions (81–83). Specifically, there is increasing evidence of a positivity effect where elderly individuals preferentially allocate their attention to and have a better memory of positive than negative/neutral stimuli (84–86); however, see (87, 88); for no positivity bias in aging]. In other words, processing of positive emotion information may be specifically important in aging and future studies should examine whether moderate HL may impact positive emotional conflicts as well.

Finally, the age of the actors in the videos could potentially be a limiting factor. Specifically, it was shown that people of different ages seem to preferentially attend to and have higher exposure to faces of their own than another age groups (89), and this may also be true for same-age voices. Considering that we used videos of younger individuals in the current study, this could be a limiting factor as older adults could process faces of younger adults differently than faces of their own age (90). Future studies should aim at controlling this factor.

## Conclusion

Age-related moderate HL changes the processing of acoustic and, potentially through compensation, visual emotional cues. As a result, people with HL may show reduced behavioral benefits for emotional stimuli in cognitive and emotional control in a multisensory environment. Importantly, such changes in multisensory integration of incongruent emotional cues may impact the emotion processing limbic network and could contribute to social isolation and depression that is sometimes observed in related to age-related HL.

## Author contributions

AZ, PK, CO, AV, ES, and SK: study design; AZ: data collection; AZ, CO, and SK: data analysis; AZ, PK, ES, AV, and SK: editing of final manuscript.

### Conflict of interest statement

The authors declare that the research was conducted in the absence of any commercial or financial relationships that could be construed as a potential conflict of interest.

## Footnotes

^1^Please find the results of an Omnibus ANOVA that compares data across both Experiments 1 and 2, adding the within-group factor of conflict type (cognitive, emotional) at the end of the results section.
