# Supplementary material for: Modulation of Cognitive and Emotional Control in Age-Related Mild-to-Moderate Hearing Loss
Source: Front Neurol. 2018 Sep 19;9:783. doi: 10.3389/fneur.2018.00783 (PMC6156531; doi:10.3389/fneur.2018.00783)
Supplement: Supplementary file 1 [file Table_1.DOCX]

Supplemental Materials

**Experiment 1**

**Timing**

There was no significant delay difference between the start of the video and the voice onset of videos between vocalizations (i.e., “A” and “O”, paired-t(7) = -1.98, *p* > 0.09, *d* = -0.115), emotions (i.e., neutral and emotional, paired-t(7) = -1.24, *p* > 0.26, *d* = -0.072), and congruence (i.e., congruent, incongruent, paired-t(7) = 0.01, *p* > 0.4, *d* = 0.001) (see Table 1). For the total video duration, there was no significant difference between vocalizations (paired-t(7) = -1.86, *p* > 0.1, *d* = -0.067) and congruence (paired-t(7) = 0.58, *p* > 0.5, *d* = 0.021). The total video duration was longer for negative than neutral videos (paired-t(7) = -3.22, *p* < 0.05, *d* = -0.116).

**Experiment 2**

Timing

There was no significant delay between the start of the video and the voice onset of videos between vocalizations (i.e., “A” and “O”, paired-t(7) = 1.49, *p* > 0.1, *d* = 0.855), emotions (i.e., neutral and emotional, paired-t(7) = 0.211, *p* > 0.8, *d* = 0.012), and congruence (i.e., congruent, incongruent, paired-t(7) = 0.420, *p* > 0.6, *d* = 0.001) (see Table 3). For the total video duration, there was no significant difference between vocalizations (paired-t(7) = 1.54, *p* > 0.1, *d* = 0.055), congruence (paired-t(7) = 0.0, *p* = 1) and emotions (paired-t(7) = 0.0, *p* = 1).

**Ratings of NH adults**

Complete videos, video streams alone, and audio streams alone were rated on a 7-point Likert scale using Self-Assessment Manikins for expressiveness, arousal, and emotion identification (Bradley & Lang, 1994) by normal hearing and hearing loss older adults (see Table 2). In the NH group, a paired-samples t-test for the complete videos showed no main effect of emotion for either arousal (paired-t(20) = 1.01, *p > 0*.3) or for expressiveness (paired-t(20) = 0.64, *p >* 0.5). The main effect of emotion was significant in the ratings of valence: participants rated emotional videos as more emotional than neutral videos (paired-t(20) = -20.67, *p* < 0.001). For the audio streams alone, the result of the analysis showed no main effect of emotion for either arousal (paired-t(20) = 1.01, *p >* 0.3) or for expressiveness (paired-t(20) = 0.65, *p >* 0.5). However, the main effect of emotion was significant for the ratings of valence (paired-t(20) = -20.84, *p <* 0.001): emotional videos were rated as more emotional than neutral videos. For the video stream alone, there was no main effect of emotion for either arousal (paired-t(20) = 0.84, *p >* 0.4) or for expressiveness (paired-t(20) = 0.55, *p >* 0.5). The main effect of valence was significant (paired-t(20) = -19.41, *p <* 0.001).

**Ratings of HL adults**

A paired-samples t-test for the complete videos showed no main effect of emotion for either arousal (paired-t(20) = 0.55, *p > 0*.5) or expressiveness (paired-t(20) = 0.64, *p >* 0.5). The main effect of emotion was significant in the ratings of valence: participants rated emotional videos as more emotional than neutral videos (paired-t(20) = -20.67, *p* < 0.001). For the audio streams alone, the result of the analysis showed no main effect of emotion for either arousal (paired-t(20) = 0.55, *p >* 0.5) or expressiveness (paired-t(20) = -0.47, *p >* 0.6). Importantly, the main effect of emotion was also not significant for the ratings of valence (paired-t(20) = -1.41, *p >* 0.17): emotional audio streams were rated as neutral. For the video stream alone, there was no main effect of emotion for either arousal (paired-t(20) = 0.62, *p >* 0.5) or for expressiveness (paired-t(20) = -0.43, *p >* 0.6). The main effect of valence was significant (paired-t(20) = -26.22, *p <* 0.001).
